# Attitudes towards complementary and alternative medicine in chronic pain syndromes: a questionnaire-based comparison between primary headache and low back pain

**DOI:** 10.1186/1472-6882-11-89

**Published:** 2011-10-07

**Authors:** Charly Gaul, Thomas Schmidt, Eva Czaja, Regina Eismann, Stephan Zierz

**Affiliations:** 1 Department of Neurology, University Duisburg-Essen, Essen, Germany; 2 Department of Neurology, Martin Luther University Halle-Wittenberg, Halle, Germany; 3 Department of Medical Psychology, Berufsgenossenschaftliche Kliniken Bergmannstrost, Halle, Germany; 4 Pain Clinic Mainz, Mainz, Germany; 5 Department of Dermatology, Martin Luther University Halle-Wittenberg, Halle, Germany

**Keywords:** Complementary and Alternative Medicine, Headache, Migraine, Low Back Pain, Motivation

## Abstract

**Background:**

Complementary and Alternative Medicine (CAM) is widely used and popular among patients with primary headache or low back pain (LBP). Aim of the study was to analyze attitudes of headache and LBP patients towards the use of CAM.

**Methods:**

Two questionnaire-based surveys were applied comparing 432 primary headache and 194 LBP patients.

**Results:**

In total, 84.75% of all patients reported use of CAM; with significantly more LBP patients. The most frequently-used CAM therapies in headache were acupuncture (71.4%), massages (56.4%), and thermotherapy (29.2%), in LBP thermotherapy (77.4%), massages (62.7%), and acupuncture (51.4%). The most frequent attitudes towards CAM use in headache vs. LBP: "leave nothing undone" (62.5% vs. 52.1%; p = 0.006), "take action against the disease" (56.8% vs. 43.2%; p = 0.006). Nearly all patients with previous experience with CAM currently use CAM in both conditions (93.6% in headache; 100% in LBP). However, the majority of the patients had no previous experience.

**Conclusion:**

Understanding motivations for CAM treatment is important, because attitudes derive from wishes for non-pharmacological treatment, to be more involved in treatment and avoid side effects. Despite higher age and more permanent pain in LBP, both groups show high use of CAM with only little specific difference in preferred methods and attitudes towards CAM use. This may reflect deficits and unfulfilled goals in conventional treatment. Maybe CAM can decrease the gap between patients' expectations about pain therapy and treatment reality, considering that both conditions are often chronic diseases, causing high burdens for daily life.

## Background

Low back pain (LBP) and primary headache disorders are frequent pain conditions at risk of chronification. Both cause a high burden of disease, large impact on social life and work capacity. The one-year prevalence of LBP in Germany is 57% for males and 66% for females [1]. The 6-month prevalence of primary headache is about 43% [2]. Whereas primary headaches often persist over decades, LBP becomes chronic only in some patients [3,4]. Standard treatment includes acute pain-relieving medication, whereas additional prophylactic medication is recommended in frequent or chronic pain. There is evidence for the effectiveness of non-pharmacological therapies like physiotherapy, aerobic exercise, cognitive behavioral therapy, progressive muscle relaxation and biofeedback in both diseases [5-8]. Both headache and LBP patients often avoid regular intake of prophylactic medication.

CAM practitioners emphasize the holistic, individualistic, empowering, and educational nature of CAM [9]. Therefore, patients are very attentive to CAM, despite the lack of evidence or possible side effects. Lifestyle factors like nutrition, smoking, consumption of alcohol, religious denomination, and income may influence frequency of CAM treatment. The use of CAM therapies increased in the population from 33.8% to 42.1% in the US from 1990 to 1997 and in Germany from 52% in 1970 to 65% in 1997 [10,11]. Likewise, the use of CAM is increasing among neurological patients [12]. Despite the growing number of publications on CAM, there is no generally-accepted definition of CAM [13]. The (US) National Center for Complementary and Alternative Medicine (NCCAM) defines CAM as "a group of diverse medical and health-care systems, practices, and products that are not currently considered to be part of conventional medicine" [14]. It was stated: "There is no alternative medicine. There is only scientifically proven, evidence-based medicine supported by solid data or unproven medicine, for which scientific evidence is lacking"[15] Therefore, strict definitions of which therapies are considered to be CAM, especially in headache or in low back pain therapy, do not exist and may depend on cultural background and health-system conditions. Patients' belief about the effectiveness of any therapy is important in the field of CAM. Some therapies have now been proven in clinical trials; others are based on traditional theories and clinical experience. Data about incidence and motivation of CAM use among headache and LBP patients in Germany are rare. The study focuses on three major questions: 1.) to evaluate the frequency of CAM use among patients with two chronic pain conditions (primary headache vs. low back pain), 2.) to compare the CAM modalities used by the two diagnostic groups, and 3.) to analyze motivation for CAM use in both diagnostic groups.

## Methods

### Study population

Four hundred forty-eight patients with primary headache according to the criteria of the International Headache Society (ICHD-II) were recruited consecutively in seven tertiary headache centers in Germany and Austria between July 2005 and December 2006 [16]. Diagnosis was established by experienced neurologists in all patients. Finally, 432 (96.4%) patients were included in the analysis [17]. Additionally, 200 inpatients suffering from low back pain (LBP), sciatica, and failed-back-surgery syndrome (FBSS) were screened in two German orthopedic departments; 194 could be included in the study. Diagnosis was established by orthopedic specialists following clinical findings. All patients attended conservative pain therapy; no patient suffered from acute LBP or underwent surgical treatment. Both studies were approved by the Ethics Committee of the Faculty of Medicine, Martin-Luther-University Halle-Wittenberg. Written informed consent was obtained from all patients.

### Research instruments

The self administered questionnaire for the two groups consisted of 42 items including socio-demographic and socioeconomic data [18], information and pain-specific data (e.g. beginning and history of disease, frequency of pain, conventional therapies, and current medication). The time for completing the questionnaire was not longer than 30 minutes. Measurement of aspects of disability and burden of disease in headache patients was done with the Migraine Disability Assessment Score (MIDAS) [19]. For LBP, the Oswestry Low Back Pain Disability Questionnaire Index (ODI) was used, which is recommended for measurement of disability. Since the ODI had not been translated and validated in German, a backward-forward translation and a feasibility study was done with 148 low-back-pain patients [20]. The questionnaire has since been validated for inpatients with chronic LBP and the German version (ODI-G) shows a very high level of test-retest-reliability and validity [21]. The study questionnaire included a list of possible CAM treatments in headaches and LBP based on own experiences and a literature search about frequently-used modalities. This was a consequence of the lack of a generally-accepted definition of CAM in both conditions. Patients could add further therapies at the end of the list. However, not all therapies were comparable because some CAM treatments are only used in headache or LBP patients (for example kinesio-taping in LBP). Patients were asked for lifetime experience with CAM. Further questions focused on attitudes towards CAM and barriers of CAM therapy. Results of both questionnaires were compared.

### Statistics

Comparisons were primarily carried out between headache and LBP patients and between patients who had versus those who had not used CAM-treatments. Frequency distributions of qualitative data were analyzed by χ^2 ^tests. Quantitative data were compared by Student's t-tests or Mann-Whitney U-tests, according to data distribution. The alpha level of significance was set at .05, two-tailed and additionally Bonferroni adjusted according to multiple comparisons. All computations were made with SPSS13.0.

## Results

### Study population

Four hundred thirty-two patients were suffering from headache and 194 from LBP. Demographic data are displayed in Table 1. Among headache patients, migraine (78.5%), tension type headache (TTH) (19.0%), combined migraine and TTH (7.4%), cluster headache or other rare headaches (2.5%), and combined cluster headache and migraine (0.5%) were reported. The LBP diagnostic group consisted of LBP (56%), sciatica (34%), and FBSS (10%). Women were more often affected by headache and LBP than men; in the headache group this is mainly due to the majority of female migraineurs. Educational level differs between headache and LBP. Headache patients report lower educational level (without vocational training) more frequently than LBP patients. Contradicting this, more headache patients than LBP patients hold a university degree. In both patient groups invalidity rate was low with no statistical difference (4% in headache vs. 6.9% in LBP).

**Table 1:** Demographic characteristics

	Headache (n = 432)	LBP (n = 194)	p value
**Gender**^a^			< .001
Male, n (%)	106 (24.5)	74 (38.1)	
Female, n (%)	326 (75.5)	120 (61.9)	
**Age**, mean (SD)^b^	40.1 (13.8)	58.2 (14.3)	< .001
**Marital status**^a^			< .001
Single, n (%)	132 (30.8)	8 (4.2)	
Partnership, n (%)	254 (59.2)	123 (64.1)	
Divorced/widowed, n (%)	43 (9.0)	61 (29.8)	
**Educational level**^a^			< .001
No vocational training, n (%)	69 (16.3)	15 (8.2)	
Vocational training, n (%)	250 (59.1)	141 (76.6)	
University degree, n (%)	93 (22)	26 (14.1)	
Other, n (%)	11 (2.6)	2 (1.1)	
**Employment statistics**^a^			< .001
Fulltime work, n (%)	202 (47.5)	60 (31.9)	
Parttime work, n (%)	81 (19.1)	13 (6.9)	
Unemployed/housewife, n (%)	93 (21.9)	22 (11.7)	
Pensioner, n (%)	32 (7.5)	80 (42.6)	
Invalidity, n (%)	17 (4)	13 (6.9)	
**Family members by the same pain condition, n (%)**^**a**^	254 (60.3)	68 (37.6)	< .001

^a^χ2 test was run; ^b ^t test was run

### Burden of disease and disability measurement

Burden of disease for both diseases is measured by the items of MIDAS and ODI and further aspects with the frequency of pain days, days with inability to work and days with reduced functioning at work as well as in social life and household (Table 2). LBP patients experienced a significantly higher burden of disease and disability than headache patients, except in regard to pain intensity which was similar in both patient groups.

**Table 2:** Burden of disease

	Headache (N = 432)	LBP (n = 194)	p value
Days with pain within the last three months (mean/SD)	30.2 (27.2)	78.2 (22.4)	< .001
Mean pain intensity (NRS 0-10/SD)	7.1 (1.8)	6.9 (2.0)	n.s.
Lost work days within the last three months due to headache/LBP (mean/SD)	4.7 (11.2)	23.2 (30.0)	< .001
Days with reduced work capacity (> 50%) within the last three months due to headache/LBP (mean/SD)	13.7 (16.9)	41.9 (36.0)	< .001
Days with reduced social capacity within the last three months due to headache/LBP (mean/SD)	9.49 (14.5)	22.67 (27.7)	< .001
Days with inability to do any housework due to headache/LBP (mean/SD)	7.6 (11.6)	20.56 (27.4)	< .001
Days with reduced function(≥ 50%) in house work within the last three months due to headache/LBP (mean/SD)	10.87 (14.9)	56.81 (31.4)	< .001

### Course of disease and treatment history

Duration of disease and duration of conventional and CAM medical treatment are displayed in Table 3. The frequency of previous use of CAM for treatment of diseases other than headache and LBP showed no significant difference between headache (18.9%) and LBP patients (13.5%).

**Table 3:** Course of disease and treatment history

	Headache	LBP	p value
Duration of disease years; mean (SD)	16.28 (13)	12.03 (12.7)	< .001
Duration of treatment years; mean (SD)	10.1 (10.1)	10.5 (11.5)	n.s.
Duration of CAM treatment for headache or LBP years; mean (SD)	7.2 (7.3)	6.3 (14.7)	n.s.
Days with analgesic medication per month; mean (SD)	8.4 (7.2)	21.1 (11.0)	< .001

### Use of CAM among headache and LBP patients

Overall, CAM was applied in 91.2% of LBP patients compared to 81.7% in the headache group. Use was similar for women (93.3% in LBP, 82.5% in headache) and men (87.8% in LBP, 79.2% in headache). Regardless of this high overall use, the difference between the two diagnostic groups was significant (p = .002). Previous experiences with CAM treatment for other diseases were low and did not significant differ between headache and LBP patients (18.9% vs. 13.5%; n.s.). The overwhelming majority of patients with previous experience of CAM use are currently CAM users for headache and LBP as well (93.6% in headache; 100% in LBP). However, previous CAM use failed to predict current CAM use when computed in a regression analysis due to the small proportion of patients in both groups not fulfilling the criteria of previous CAM use. The distribution of different CAM treatments is displayed in Table 4.

**Table 4:** Frequencies of used CAM treatments

	Headache (n = 353)	LBP (n = 177)	p value
Pharmacological CAM			
High-dose megavitamins; n (%)	32 (9.1)	40 (22.7)	< .001
Herbal medicine; n (%)	24 (6.8)	23 (13.1)	n.s.
Homeopathy; n (%)	100 (28.3)	21 (11.9)	< .001
Non-pharmacological CAM			
Transcutaneous electrical nerve stimulation (TENS); n (%)	81 (22.9)	28 (15.8)	n.s.
Thermotherapy (Fango); n (%)	103 (29.2)	137 (77.4)	< .001
Acupuncture; n (%)	252 (71.4)	91 (51.4)	< .001
Manual Therapy; n (%)^b^	66 (18.7)	80 (45.2)	< .001
Massages; n (%)	199 (56.4)	111 (62.7)	n.s.
Magnetic field; n (%)	0	29 (16.4)	< .001
Kinesio-taping; n (%)	^a^	10 (5.6)	
Osteopathy; n (%)	^a^	10 (5.6)	
Traditional Chinese medicine; n (%)	41 (11.6)	15 (8.5)	n.s.
Others^b^	46 (13)	31 (17.5)	n.s.

^a ^not applicable

^b ^(e.g. diets, music therapy, oxygen therapy, psychofonia, meditation, climate therapy); n (%)

The relationship between the burden of disease and frequency of CAM use was calculated. Due to overall severe disability in the patient populations (64.2% MIDAS severity score IV and 39.4% ODI-Score > 40) in both patient groups, no significant differences regarding CAM use could be detected.

### Attitudes towards using CAM treatment

**Figure 1:**
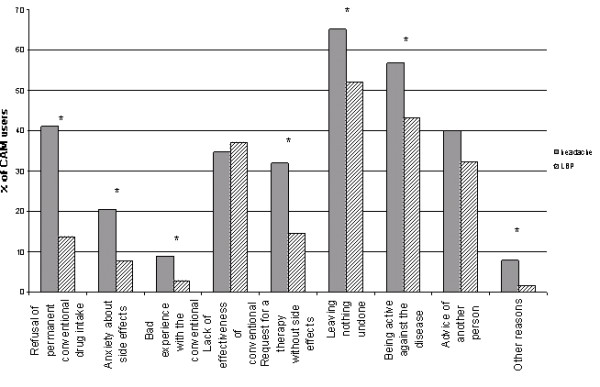
**Motivation for CAM use among headache and LBP patients**. Reasons for CAM use given by patients with headache and LBP are shown. LBP: low back pain; *significant; χ2 tests was run, multiple answers were appropriate.

Reasons for CAM use differ between headache and LBP patients (Figure 1). Reasons for not using CAM given by the 79 non-using headache patients were 'not heard about it' (12.7%), 'I do not believe in this' (8.9%), 'no interest' (3.8%), other reasons (25.3%); 21% declared no reasons at all for non-use. The non-CAM using group in LBP consisted of only 17 patients. Only one patient gave the reason 'too expensive', none of the others gave any specific reason.

### Additional factors which may influence CAM use

Three hundred twenty patients reported membership in a church (93 Lutheran, 206 Catholic, 19 others) while 272 did not. No significant association with CAM use was found for membership in a church. CAM users are more likely to live with a partner. 3.4% of the headache and 1.8% of the LBP patients are vegetarians (n.s.). Due to the small number, it is not possible to determine if this lifestyle factor predicts CAM use. Additional lifestyle information and factors which may influence CAM use are displayed in Table 5. More patients using CAM do not drink alcohol compared to non-users, whereas no statistical differences were found for occasional and daily alcohol consumption. Satisfaction with therapy was analyzed among non-CAM and CAM users (Table 6).

**Table 5:** Additional lifestyle information and factors which may influence CAM use

	Non-CAM	CAM	p value
Never drinking alcohol; n (%)	19 (20.7)	173 (33.8)	n.s
Non-smoker; n (%)	47 (50.5)	374 (73.2)	< .001
Sports			n.s
-never; n (%) -once or more a week; n (%)	31 (36.5) 54 (63.5)	142 (29.5) 340 (70.5)	

**Table 6:** Satisfaction with current conventional therapy

Measure	Non-CAM (n = 96)	CAM (n = 530)	p value
Satisfaction with conventional medical therapy			n.s
Very satisfied; n (%)	22 (28.9)	139 (29.4)	
Moderately satisfied; n (%)	30 (39.5)	229 (48.5)	
Not satisfied; n (%)	24 (31.6)	104 (22.0)	

## Discussion

The high overall CAM use (87%) despite substantial differences in disease-specific aspects of the two patient populations revealed in the study is in line with population-based findings showing frequent CAM use in LBP (57%), cold (29%), headaches (19%), strain (15%) and gastrointestinal ailments (12%) [22].

LBP and headache patients differ regarding demographic data (Table 1), reflecting the characteristics of the diseases (e.g. higher age and later onset in LBP), which also can influence lifetime experience with CAM. The educational level differs significantly with a higher portion holding a university degree among headache patients. This may have two reasons: 1. LBP patients are older and education levels increased during the last decades 2. Patients with lower education are likely to do manual work and therefore suffer more from LBP. Duration of disease is significantly longer for headache patients due to the onset of primary headache in adolescence, whereas LBP mostly begins in middle-aged or older people.

Patients with LBP were more likely unable to work than headache patients. Headache patients suffer most from episodic headache with only half the pain days per month compared to LBP. Therefore, the intake of analgesics in LBP is more than twice as high as for headache patients. However, there were no differences in mean pain intensity in the two groups. The significantly higher number of affected family members in headache compared to LBP (Table 1) reflects the genetic background of primary headaches.

Chronic pain was found to double the odds of using CAM [23]. A recent Canadian survey found migraine and asthma as predictors for higher CAM use in the population. However, this was not supported for other chronic diseases with a rather constant nature of symptoms (significantly lower CAM use in diabetes, no significant rate of CAM use compared to general population in epilepsy) [24].

Conventional treatment often does not result in resolution of symptoms; therefore patients look for alternative treatment options. In 2002, a population-based US survey revealed that 6% of the population use CAM for treatment of LBP [25]. In our study, acupuncture, massages, and thermotherapy are the most frequent CAM therapies in headache and in reverse order for LBP. The most frequently used CAM therapies in Germany are exercise therapy, herbal medicine, hydrotherapy, medical massage, homeopathic remedies and acupuncture [22]. Considering the different therapeutic impact of physiotherapy in headache and LBP, physiotherapy was not defined as a CAM modality. Some specific techniques are recommended in treatment guidelines especially for LBP. However, the evidence for physiotherapy and endurance sports is weak in headache treatment while more evidence is found in LBP [6,26,27]. There is no evidence supporting the use of transcutaneous electrical nerve stimulation (TENS) in headache or LBP therapy [5,28].

Understanding the high frequency of CAM use leads to an analysis of motivations and barriers to treatment. Prior CAM use was associated with current CAM use, showing the adherence to previous experience and behavior. 'Lack of effectiveness of conventional treatment' was given as the strongest motivational reason for CAM treatment. This is not surprising regarding clinical trials in headache prophylaxis showing a 50% reduction of headache days in only half of the patients. Similarly discouraging data are reported for treatment of LBP. Effects of acute pain medication as well as of prophylactic medication were estimated only as "moderate" in a recent review [27]. Contradicting this, dissatisfaction with conventional treatment was not significantly associated with more frequent CAM use in our study. However, patients expect an improvement in the chronic condition. CAM therapies are estimated as helpful by most patients and they would like to have more information on CAM and would appreciate prescriptions of CAM [22]. Headache patients raised three times more concerns about permanent conventional drug intake than LBP patients. This could be a consequence of the longer duration of disease and repeated frustration about past treatments. Notably, concerns about drugs are four times more frequent in both groups than concerns resulting from bad experience with conventional medical practitioners.

In addition to the motivational factors, barriers for CAM use could be asked among the patients who had no experience with CAM. In the present study, the substantial number of patients offering no declaration of the given reasons in the questionnaire is striking (21.5% in headache; 82.4% in LBP), implying that possible reasons for not having used CAM are not sufficiently shown in the predetermined answer alternatives.

Apprehension of side effects is more important for headache than for LBP patients, but is a substantially itemized pro for CAM in both. This reflects the general assumption of many patients that CAM has no or fewer side effects. However, numerous herbal remedies have a potentially sensitizing capacity for allergic contact dermatitis and lead to IgE-mediated clinical symptoms or may have carcinogenic properties. Interaction between conventional drug treatment and CAM has to be considered. Injuries resulting in pneumothorax, cardiac tamponade or spinal injury and infectious complications are rare side effect of acupuncture [29].

To be active against the pain condition is reported as a motivation for CAM therapy more frequently in headache than in LBP patients. Furthermore, the wish to "leave nothing undone" was displayed clearly in both groups. The patients' activities in this point may reflect the pain intensity and frequency, the high burden of disease and unsatisfactory treatment experiences. However, no significant difference was found between CAM and non-CAM users regarding satisfaction with conventional pain treatment. This contradicts studies revealing an association between treatment satisfaction and search for CAM [30].

An important point is the advice of other persons to use CAM, which increases CAM use in both LBP (32.2%) and headache (40%) patients. This may correspond to family traditions or experience relayed from friends. Only a small group of patients gives other reasons for CAM treatment, reflecting the accuracy of the questionnaire to capture the relevant information. Other sources of information about CAM treatment are television, newspapers and internet. The gathering of more information from media coverage by the headache patients may reflect the lower mean age.

Cost and reimbursement from insurance may influence CAM use, too. However, only a small portion of the non-users among the headache (12.7%) and LBP (5.9%) patients give the cost as a barrier against CAM use. The situation of the statutory health insurance system in Germany should also be considered. For regular treatment, all costs are paid by the insurance directly without the need for prior administrative decision (including massages, homeopathic remedies and acupuncture in LBP but not in headache).

It might be helpful to ask patients about current CAM use to avoid side effects and pharmacological interactions. Their understanding of their treatment wishes and beliefs should also be taken into consideration. CAM therapies are popular especially among general practitioners in Germany; therefore it is not surprising that 71.7% of the headache and 77.4% of the LBP patients had talked about CAM use with their physicians.

As a limitation, the survey was performed among headache outpatients treated in tertiary centers and among inpatients with LBP. This population may be characterized through higher disability and burden of disease compared to patients of the general population. This could influence the frequency and motivation of CAM use. In future studies, we suggest using a more strict definition of CAM therapies and asking exactly the same questions in conditions to be compared. The specific healthcare system should be considered in comparing results from different countries. Reimbursement by the health care insurance might be important as an additional predictor of CAM use.

## Conclusion

Despite disease-specific differences in LBP and in headache patients, both groups show high use of CAM mostly in conjunction with conventional treatment with only slight specific differences in preferred methods and motivation for CAM use. This may reflect deficits and unfulfilled goals in conventional treatment of chronic diseases associated with pain conditions. Clinicians should ask patients about their CAM use, discussing benefits and possible risks. Understanding motivation for CAM treatment is important for communication with patients about CAM and conventional treatment, because motivation derives from wishes for non-pharmacological treatment, to be more involved in treatment and avoid side effects. Maybe CAM can decrease the gap between patients' expectations on pain therapy and current treatment reality, keeping in mind that headache and LBP are chronic diseases with high burden on daily life for many people.

## Competing interests

The authors declare that they have no competing interests.

## Authors' contributions

All authors made a substantial contribution to this work. CG, TS and EC conceived and designed the study. EC and RE obtained the data, CG and TS conducted the analysis and drafted the manuscript. SZ carefully revised the manuscript. All authors participated in the interpretation of data, revised the manuscript and approved the final version of the manuscript that is now being submitted for publication.

## Pre-publication history

The pre-publication history for this paper can be accessed here:

http://www.biomedcentral.com/1472-6882/11/89/prepub
